# A Porous Cobalt (II) Metal–Organic Framework with Highly Efficient Electrocatalytic Activity for the Oxygen Evolution Reaction

**DOI:** 10.3390/polym9120676

**Published:** 2017-12-06

**Authors:** Qingguo Meng, Jianjian Yang, Shixuan Ma, Mujun Zhai, Jitao Lu

**Affiliations:** 1 College of Chemical Engineering and Environmental Chemistry, Weifang University, Weifang 261061, China; mengqg@wfu.edu.cn (Q.M.); jitaolu@wfu.edu.cn (J.Y.); wfumqg@163.com (S.M.); 2 The Testing Center of Shandong Bureau of China Metallurgy and Geology Bureau, Jinan 250014, China; zhaimujunyangjia@163.com

**Keywords:** cobalt, metal-organic frameworks, Wei topology, oxygen evolution reaction

## Abstract

A 3D porous framework (**[Co_1.5_(tib)(dcpna)]·6H_2_O**) (**1**) with a Wei topology has been synthesized by solvothermal reaction of 1,3,5-tris(1-imidazolyl)-benzene (tib), 5-(3′,5′-dicarboxylphenyl)nicotinic acid (H_3_dcpna) and cobalt nitrate. The electrocatalytic activity for water oxidation of **1** has been investigated in alkaline solution. Compound **1** exhibits good oxygen evolution reaction (OER) activities in alkaline solution, exhibiting 10 mA·cm^−2^ at η = 360 mV with a Tafel slope of 89 mV·dec^−1^. The high OER activity can be ascribe to 1D open channels along b axis of **1**, which expose more activity sites and facilitate the electrolyte penetration.

## 1. Introduction

Electrochemical splitting of water into H_2_ and O_2_ is one of the effective and environmentally friendly methods for a possible hydrogen economy [1,2,3,4,5]. The water electrolysis is composed of two half reactions: the hydrogen evolution reaction (HER, 2H^+^ + 2e^−^ → H_2_ in acidic electrolytes) and the oxygen evolution reaction (OER, 4OH^−^ → 2H_2_O + 4e^−^ + O_2_ in alkaline electrolytes) [6,7,8,9]. OER is a demanding step which involves multiproton-coupled electron-transfers and oxygen–oxygen bond formation. Hence, OER has a high activation energy barrier and requires a higher energy (higher overpotential) to overcome the kinetic barrier [10,11,12]. To facilitate energy storage and conversion systems, such as batteries, supercapacitors and water splitting, plenty of efficient and stable OER electrocatalysts have been exploited [13,14,15]. Among which, precious-metal oxides such as IrO_2_ and RuO_2_ are well known as the most active electrocatalysts for the OER in both acidic and alkaline electrolytes due to their low overpotential and small Tafel slope [16,17,18,19]. However, both are made of precious metals which render them unsuitable for use in largescale practical applications. Therefore, substantial research effort has been devoted to explore non-noble metal catalysts with high electrocatalytic activity and stability, such as 3d-transition-metal oxides, perovskites, transition-metal sulfides, hydro(oxy)oxides, phosphates, non-metal compounds and molybdates, along with various molecular catalysts [20,21,22,23,24,25,26]. Among these well-developed electrocatalysts, 3d-transition metal compounds, especially cobalt-based compounds, have attracted growing research interests and have exhibited high electrocatalytic activity for OER due to their various redox properties and unusual capability to form high-oxidation cobalt species during OER process [27,28].

As a new type of porous material, porous metal–organic frameworks (MOFs), which are built from metal-based nodes and organic linkers, are often considered as ideal alternatives in important catalytic processes owing to their inherent features, such as large surface area, unique porosity and tailorable functionality [29,30,31,32,33,34]. In particular, some reports show that MOFs possess high electrocatalytic activity for OER owing to its high surface areas, which exposed more active site. For example, Sun’s group have reported a 3D non-interpenetrating porous metal-organic framework [Pb_2_(H_2_TCPP)]·4DMF·H_2_O (Pb-TCPP) (H_6_TCPP = 5,10,15,20-tetra(carboxyphenyl)porphyrin, DMF = (*N*,*N*-dimethylformamide), which shows electrocatalytic activity in alkaline electrolytes [35]. However, lead is a kind of toxic metal, which is not desired in practical applications. Wang’s group synthesized a highly stable Fe/Ni metal–organic framework by electrochemical deposition. This mixed-metal MOF film-based electrode shows high activity with low overpotential of 270 mV at 10 mA·m^−2^, high current densities and a small Tafel slope of 47 mV·dec^−1^ [36]. Despite there are numerous advantages of MOFs used as electrocatalysts for OER, the directly use of MOFs for electrocatalytic OER is still in its infancy.

In the present work, a porous metal–organic frameworks (**[Co_1.5_(tib)(dcpna)]·6H_2_O**) (**1**) based on 1,3,5-tris(1-imidazolyl)-benzene (tib) and 5-(3′,5′-dicarboxylphenyl)nicotinic acid (H_3_dcpna) has been synthesized and characterized. Single-crystal X-ray diffraction analysis reveals that **1** exhibits a non-penetrated 3D porous framework. The electrocatalytic activity for water oxidation of **1** has been investigated in alkaline solution. Compound **1** exhibits a good oxygen evolution reaction (OER) activities at a low overpotential of 360 mV for 10 mA·cm^−2^ current density with a Tafel slope of 89 mV·dec^−1^ in 1 M KOH.

## 2. Experimental

### 2.1. Materials and Methods

All chemicals were of reagent grade and purchased from commercial vendors without further purification. Elemental analyses were recorded on a Perkin-Elmer 240 elemental analyzer(PerkinElmer, Inc., Billerica, MA, USA). Powder X-ray diffraction (PXRD) measurements were conducted with a Bruker AXS D8 Advance instrument (Karlsruhe, Germany). The Fourier Transform Infrared (FT-IR) spectra were recorded on a Nicolet 330 FTIR Spectrometer (Nicolet Instrument Inc., Madison, WI, USA) in the range of 4000–400 cm^−1^ using the KBr pellet method. TGA experiments were carried out on a Perkin-Elmer TGA 7 instrument (PerkinElmer, Billerica, MA, USA) with heating rate of 10 °C min^−1^ under nitrogen stream. All electrochemical measurements were performed on a CHI760E electrochemical workstation (Chenhua Instrument Shanghai Co., Ltd., Shanghai, China).

### 2.2. Synthesis of **[Co_1.5_(tib)(dcpna)]·6H_2_O** (***1***)

A mixture of tib (0.018 mmol, 5 mg), H_3_dcpna (0.023 mmol, 6.6 mg) and Co(NO_3_)_2_·6H_2_O (0.034 mmol, 10 mg) were dissolved in 6 mL of mixed solvents of DMF/1,4-dioxane/H_2_O (*v*/*v*/*v* = 2.5:1:1). After performing an ultrasound at room temperature for 10 min, the glass tube was sealed and placed in an oven and slowly heated to 80 °C from room temperature in 700 min, kept at 80 °C for 3 days and slowly cooled to 35 °C in 1000 min. Then, the glass tube was slowly heated to 120 °C in 700 min and kept at 120 °C for 3 days. After cooling down to room temperature in 1000 min, violet block-shaped crystals suitable for X-ray diffraction analysis were separated by filtration with the yield of 8.853 mg, 51.6% (based on cobalt). Anal. Calc. (found) for C_29_H_40_O_12_N_7_Co_1.5_: C, 45.41 (45.34); H, 5.26 (5.71), N 12.78 (13.24) IR (KBr): m (cm^−1^) = 3434 (w), 1614 (s), 1542 (s), 1513 (s), 1438 (s), 1409 (m), 1369 (m), 1294 (w), 1261 (m), 1116 (w), 1074 (m), 1010 (m), 856 (w), 777 (m), 734 (m), 651 (m), 4417 (w).

### 2.3. X-ray Crystallography

The unit cell determinations and data collections for the Single crystal of **1** were performed on a Bruker D8 Apex II Image Plate single-crystal diffractometer. The data were collected using a graphite monochromated Mo Kα radiation source (*λ* = 0.71073 Å) at 293 K. All absorption corrections were performed by the multi-scan program SADABS [37]. In all cases, the highest possible space group was chosen. The structure was solved by direct methods and refined on *F*^2^ by full-matrix least-squares procedures with SHELXL-97 program package [38,39]. As no appropriate disorder model can be used to deal with the highly disordered solvent molecules, the SQUEEZE subroutine of the PLATON software suit was used to remove scattering from the disordered solvent molecules [40]. The following new files were used to further refine the structures. The Crystal data and structure refinement for **1** are listed in Table 1. Selected bond lengths and angles for **1** are summarized in Table 2. The Cambridge Crystallographic Data Centre (CCDC)-1579569 contains the supplementary crystallographic data for this paper. These data can be obtained free of charge at www.ccdc.cam.ac.uk/conts/retrieving.html or from the Cambridge Crystallographic Data Centre, 12, Union Road, Cambridge CB2 1EZ, UK; fax: (internat.) +44-1223/336-033; email: deposit@ccdc.cam.ac.uk.

### 2.4. Experiment for Electrochemical Measurements

A glassy carbon electrode (GCE) with the diameter of 3.0 mm (Chenhua Instrument Shanghai Co., Ltd., China) was used as work electrode. Before electrochemical test, the surface of the GCE was rubbed carefully with alumina slurry and rinsed with ethanol and deionization water successively. A conventional three-electrode system was used with Saturated calomel electrode (SCE) as the reference electrode and Platinum (Pt) foil as counter electrode (surface area of 15 mm × 15 mm). The potential values are corrected to the reverse hydrogen electrode (RHE) according the equation E (RHE) = E (SCE) + 0.245 + 0.0591 pH V. Typically, 5.0 mg of **1** and 20.0 μL Nafion solution (5 wt %) were dispersed in 1 mL of mixed solvents of deionized water and ethanol (*v*/*v* = 1:1) to form a homogeneous solution. Then, 10.0 μL of the homogeneous solution was put on a glassy carbon electrode to prepare the work electrode. The geometric area of GCE is 0.07069 cm^2^. To achieve a constant state of the anodic electrode, the catalyst was electrochemically pre-activated by 15 cyclic voltammetry scans at a scan rate of 10 mV·s^−1^ before the electrochemical test. Linear sweep voltammogram curves were examined in 2 M KOH (pH = 14.3), 1 M KOH (pH = 14.0) and 0.1 M KOH (pH = 13.0) for electro-catalysts, respectively. The scan rate is 10 mV·s^−1^ and the scan region ranges from 0 to 0.9 V vs. SCE.

### 2.5. Calculation Method of the Turnover Frequency (TOF)

TOF was calculated with Equation (1):

(1) $$\mathrm{TOF}=\frac{jA}{4nF}$$

where *j* is the measured current density (mA·cm^−2^) at a measured overpotential in the range of 343 mV to 393 mV, *A* is the geometric area of the GCE, *n* is the mole number of the coated catalysts, and *F* is the Faraday constant (96,500 C·mol^−1^).

## 3. Results and Discussion

### 3.1. X-ray Single Crystal Structure

**Figure 1:**
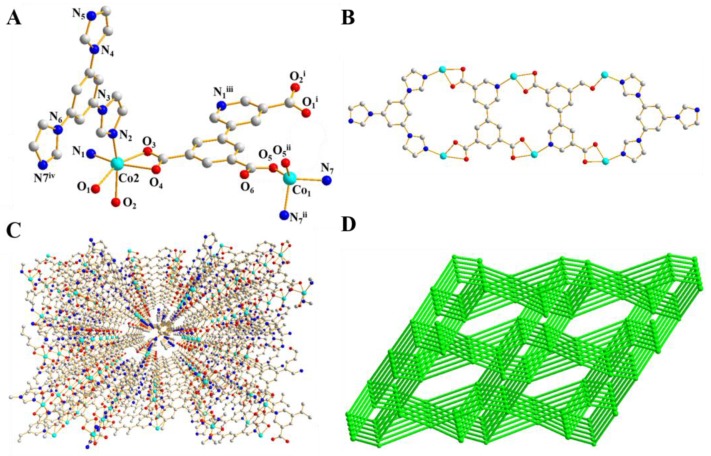
(**A**) The coordination environment of two Co(II) ions; (**B**) coplanar structure with two 21-membered-rings and one 18-membered-ring; and (**C**,**D**) the 3D structure and simplified 4-connected Wei topology of **1**.

X-ray single-crystal diffraction analysis reveals that **1** possesses a neutral three-dimensional metal–organic framework with Wei topology, which is rare in the literature. It is crystallized in monoclinic *C*2/*c* space group and there are one and a half Co(II) ions, one dcpna ligand, and one tib ligand in the asymmetrical unit of **1**. The coordination environment of both of the two Co(II) ions are shown in Figure 1A. As can be seen, Co1 is tetra-coordinated with two carboxylate oxygen atoms (Co1-O5 1.993(3) Å) from two different dcpna ligands and two N atoms of two different tib ligands (Co1-N7 2.047(3) Å), forming a distorted tetrahedron geometry, while Co2 center has a distorted octahedral geometry. As depicted in Figure 1A, Co2 is six-coordinated by four carboxylate oxygen atoms and one N atom from three dcpna ligands and one N atom from a tib ligand. The bond distances of Co-O vary from 2.059(2) Å to 2.296(3) Å, the distances of Co-N are 2.092(3) Å and 2.054(3) Å, which are comparable with those for other reported Co compounds [41,42]. In **1**, in combination with two μ_2_-tib, two μ_4_-dcpna ligands whose three carboxylate groups adopt μ_1_-η^1^-η^1^ and μ_1_-η^1^-η^0^ coordination mods link four Co(II) ions to form a coplanar structure with two 21-membered-rings and one 18-membered-ring (Figure 1B). Then, the neighbor coplanar structures connected to each other to furnish an open 3D frameworks through the coordination of carboxylate groups of dcpna ligand and Co(II) ions (Figure 1C). The structure shows small 1D channels along b axis, in which the disordered solvent molecules reside. The approximate channel sizes for **1** is 3.9 × 5.1 Å^2^. The percent void volume obtained using the PLATON software is 30.4% in the 3D networks of **1**. 

The topological method is used to simplify the structure of **1**, Figure 1D. Topologically, both Co(II) ion and dcpna ligand can be seen as a 4-connected node, and then the overall 3D framework can be rationalized as a 4,4-conected network with the point symbols {3·4·6·7·8^2^}4{3^2^·6^2^·7^2^} and belongs to a Wei topology.

### 3.2. Electrocatalytic Activity

As mentioned above, a myriad of previous reports have indicate that MOFs always show high catalytic activity in plenty of catalytic processes due to their large surface area, unique porosity, and tailorable functionality. However, MOFs used as OER catalyst are seldom reported. Therefore, to test the catalytic performance of **1** for electrochemical oxidation of water to dioxygen, **1** was loaded onto a glassy carbon electrode using Nafion as binder to investigate the electrocatalytic activity of **1** in alkaline electrolytes.

**Figure 2:**
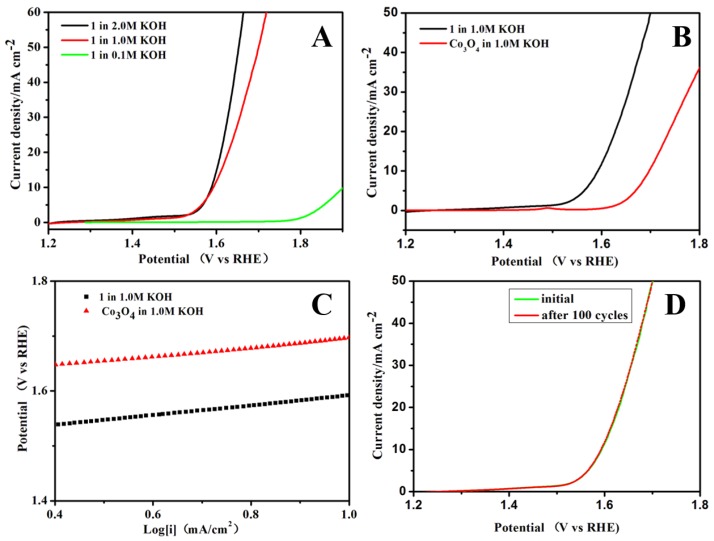
(**A**) Polarization curves of **1** in 0.1 M, 1 M and 2 M KOH; (**B**) polarization curve of **1** and benchmark Co_3_O_4_ in 1 M KOH; (**C**) polarization curve derived Tafel plots of **1** and benchmark Co_3_O_4_ in 1 M KOH; and (**D**) polarization curves for the 1st and 100th potential cycles of **1** in 1 M KOH.

**Figure 3:**
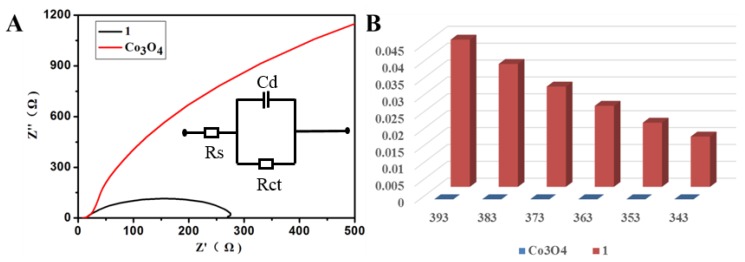
(**A**) Nyquist plots of **1** and benchmark Co_3_O_4_ examined at 0.5 V (vs. Saturated calomel electrode SCE); and (**B**) TOFs of **1** and benchmark Co_3_O_4_ at different overpotentials from 343 to 393 mV.

Figure 2A gives LSV curves of **1** in an O_2_-saturated 0.1, 1.0 and 2 M KOH solution with a scan rate of 10 mV·s^−1^. As can be seen, the electrocatalytic OER activity of **1** significantly increases with enhancing the concentration of KOH solution, indicating that a high concentration of KOH is beneficial for the improvement of OER performance, which is in good agreement with previous observations in other electrocatalytic systems [35,43,44]. As a comparison, Co_3_O_4_, purchased fromAlfa Aesar(China) Chemicals Co., Ltd., Shanghai, China, was loaded onto a glassy carbon electrode to test the electrocatalytic activity in the same conditions. Figure 2B shows the LSV curves of the different catalysts in 1.0 M KOH solution with a scan rate of 10 mV·s^−1^. The OER onset potential of **1** is estimated to be about 1.51 V (vs. RHE), which is earlier than that of the benchmark Co_3_O_4_ counterpart (1.61 V vs. RHE). For practical purposes, one is required to apply a high overpotential in order to have significant magnitude of current density. The overpotential required to reach a current density of 10 mA mA·cm^−2^ (η_10_) is chosen because it is a metric associated with solar fuel synthesis. As can be seen in Figure 2B, **1** delivers a current density of 10 mA·cm^−2^ at an overpotential of 1.59 V, which is observed to be smaller than that of Co_3_O_4_ (1.70 V). It is worth pointing out that the η_10_ value for **1** compares recently reported state of the art Co-based and MOF-based OER catalysts (Table 3) [45,46,47,48,49,50,51,52,53,54,55,56]. Tafel plots was always employed to estimate the electrocatalytic kinetics. As shown in Figure 2C, the Tafel slope of **1** is 89 mV·dec^−1^ in 1 M KOH, which is bigger than that of Co_3_O_4_ (73 mV·dec^−1^), and comparable to that of the previously reported highly active OER catalysts (Table 3) [45,46,47,48,49,50,51,52,53,54,55,56]. Durability is of great importance for the practicability of catalyst electrode. The stability of **1** was examined by continuous cyclic voltammetry (CV) scanning between 1.38 and 1.78 V·vs. RHE in 1 M KOH solution at a scan rate of 100 mV·s^−1^. Interestingly, after 100 cycles, no obvious changes in current density is observed from LSV curves indicating a long-term stability of **1** in OER processes (Figure 2D). Electrochemical impedance spectroscopy (EIS) was performed to further estimate the kinetics of the catalysts at the electrode and electrolyte interface during the OER process. Figure 3A describes the obtained Nyquist plots of **1** and Co_3_O_4_, respectively. Both **1** and Co_3_O_4_ were fitted using the same equivalent circuit, which contained three components: charge-transfer resistance (Rct), solution resistance (Rs), and constant-phase resistance (Rcp). The charge transfer resistance (Rct) at the surface of the catalysts can be confirmed by the diameter of a semicircle at high frequencies in the Nyquist plot. Generally, small Rct value means fast OER kinetics [28,57]. Compared with Co_3_O_4_ in 1.0 M KOH, the Rct values of **1** keep the smaller Rct value in 1 M KOH. Hence, such a low Rct value of **1** indicates its high charge transfer process at the surface of **1** (Table 4). The intrinsic activity of catalysts were also further evaluated by the turnover frequency (TOF) from 343 to 393 mV. As depicted in Figure 3B, the TOF on **1** is apparently higher than those of benchmark catalyst Co_3_O_4_ demonstrating the better intrinsic OER activity of **1**. 

### 3.3. X-ray Powder Diffraction Analyses, IR Spectra and Thermal Analyses

**Figure 4:**
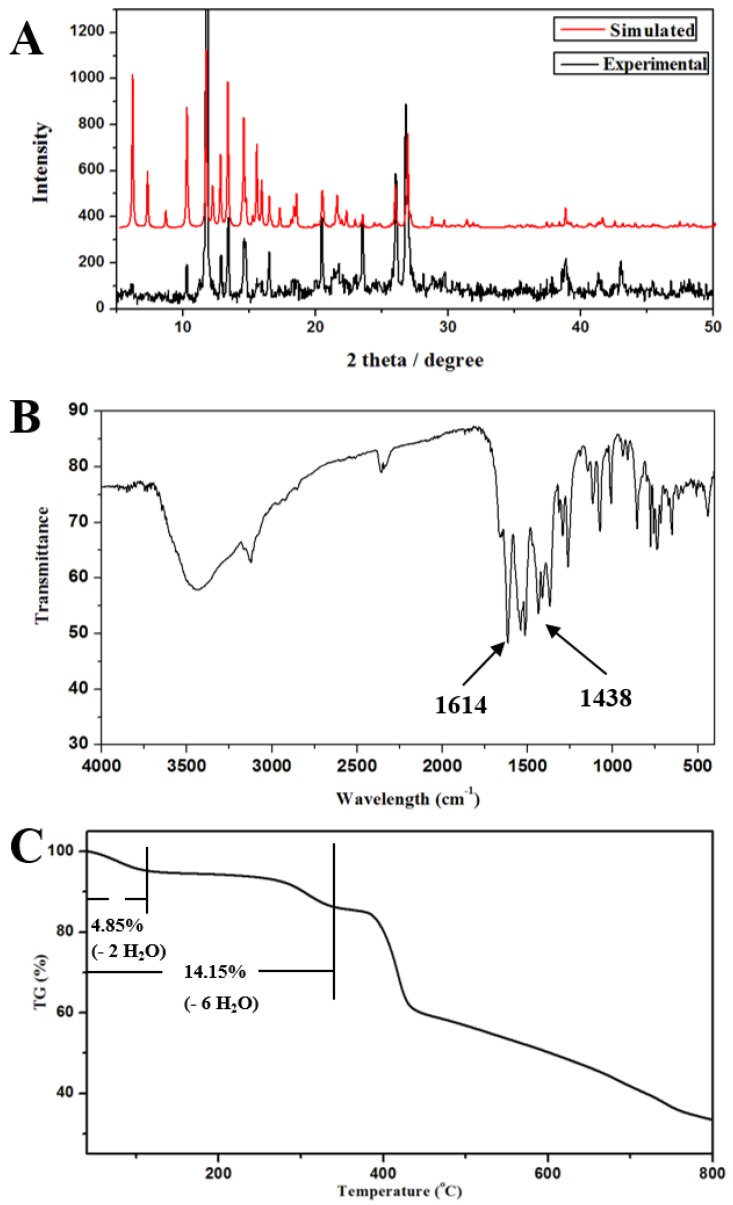
(**A**) FT-IR spectrum of **1**; (**B**) the powder PXRD pattern and the simulated one from the single-crystal diffraction data of **1**; and (**C**) TGA curve of **1**.

The PXRD pattern was applied to confirm the phase purity of **1** (Figure 4A). The simulated XRD pattern for **1** was acquired from the crystal data using mercury software. As can be seen, most of the peak positions of simulated are in good agreement with the experimental patterns, indicating the high purity of the samples. 

FT-IR spectrum of **1** was also investigated (Figure 4B). As indicated by the IR spectroscopy result of **1**, the sharp bands observed at about 1614 and 1438 cm^−1^ can be ascribed to symmetric and asymmetric stretching vibrations of carboxylic group, respectively [58,59].

To evaluate the thermal stability and their structural variation with the temperature, TGA of **1** was tested under a N_2_ atmosphere using polycrystalline samples (Figure 4C). Compound **1** has three identifiable weight loss steps: The first weight loss before 114 °C is consistent with the removal of two lattice H_2_O molecules (obsd 4.85%, calcd 4.76%). The second one can be ascribe to the removal of four lattice H_2_O molecules (obsd 14.15%, calcd 14.28%), which is in the range of 114–348 °C. The third one from 348 to 442 °C is attributed to the collapse of the framework.

## 4. Conclusions

In summary, a 3D porous framework with a Wei topology has been synthesized by solvothermal reaction of tib, H_3_dcpna and cobalt nitrate. The electrocatalytic activity for water oxidation of **1** has been investigated. Compound **1** shows good oxygen evolution reaction (OER) activities in alkaline solution, exhibiting 10 mA·cm^−2^ at η = 360 mV with a Tafel slope of 89 mV·dec^−1^, and excellent cycling stability, which is superior to the standard Co_3_O_4_ counterpart. The high OER activity can be ascribed to 1D open channels along b axis of **1**, which expose more activity sites and facilitate the electrolyte penetration.

## Figures and Tables

**Table 1:** Crystal data and structure refinement for **1**.

Complex	1
Empirical formula	C_29_H_18_Co_1.5_N_7_O_6_
Formula weight	648.90
Temperature/K	293(2)
Crystal system	monoclinic
Space group	*C2/c*
*a*/Å	35.366(7)
*b*/Å	9.2025(18)
*c*/Å	25.003(5)
*α*/°	90.00
*β*/°	124.34(3)
*γ*/°	90.00
Volume/Å^3^	6719(2)
*Z*	8
*ρ_calc_*g/cm^3^	1.283
*μ*/mm^−1^	0.797
*F*(000)	2636.0
Crystal size/mm^3^	0.29 × 0.25 × 0.18
Radiation	MoKα (*λ* = 0.71000)
*2Θ* range for data collection/°	6.08 to 54.9
Index ranges	−45 ≤ *h* ≤ 45, −11 ≤ *k* ≤ 9, −32 ≤ *l* ≤ 31
Reflections collected	31073
Independent reflections	7671 [*R_int_* = 0.0698, *R_sigma_* = 0.0717]
Data/restraints/parameters	7671/0/393
Goodness-of-fit on *F*^2^	1.012
Final *R* indexes [I ≥ 2*σ* (I)]	*R*_1_ = 0.0547, *wR*_2_ = 0.1418
Final *R* indexes [all data]	*R*_1_ = 0.0959, *wR*_2_ = 0.1685
Largest diff. peak/hole/e Å^−3^	0.53/−0.58

**Table 2:** Selected bond lengths (Ǻ) and angles (˚) for **1**.

Co1-O5	1.993(3)	Co1-N7	2.047(3)	Co2-O3	2.059(2)
Co2-O1	2.128(2)	Co2-O4	2.296(3)	Co2-O2	2.194(3)
Co2-N1	2.092(3)	O5 ^1^-Co1-O5	126.18(17)	O5-Co1-N7 ^1^	108.31(12)
O5 ^1^-Co1-N7 ^1^	106.78(12)	N7 ^1^-Co1-N7	96.47(17)	O3-Co2-O1	157.03(12)
O3-Co2-O4	60.35(9)	O3-Co2-O2	101.56(11)	O3-Co2-N1	96.76(11)
O1-Co2-O4	103.00(10)	O1-Co2-O2	60.64(10)	O2-Co2-O4	90.64(11)
N1-Co2-O1	98.04(11)	N1-Co2-O4	156.94(10)	N1-Co2-O2	91.53(12)
N2-Co2-O3	97.93(11)	N2-Co2-O1	97.79(11)	N2-Co2-O4	90.13(11)
N2-Co2-O2	157.97(11)	N2-Co2-N1	96.31(12)		

^1^ 1 − X, +Y, 1/2 − Z.

**Table 3:** Comparison of OER catalytic performance of recently reported state of the art Co-based and MOF-based OER catalysts.

Catalysts	Overpotential@10 mA mA·cm^−2^ (mV)	Tafel slope (mV·dec^−1^)	Electrolyte	Substrate	References
MAFX27-OH	387	60	1 M KOH	Glassy Carbon	[45]
UTSA-16	410	40	1 M KOH	Glassy Carbon	[43]
Co-ZIF	510@1 mA·cm^−2^	193	pH = 13.4	FTO	[46]
Co-TpBpy	400@1 mA mA·cm^−2^	/	pH = 7.0	Glassy Carbon	[47]
CoTPyP	400@1 mA mA·cm^−2^	/	0.1 M NaOH	FTO	[48]
Co-WOC-1	390@1 mA mA·cm^−2^	128	0.1 M KOH	Au(111) single-crystal	[49]
Co_3_O_4_/NRGO	420	83	1 M KOH	Glassy Carbon	[50]
hollow Co_3_O_4_ microtubes	290	84	1 M KOH	Ni foam	[51]
Co_3_O_4_ nanosheets	300	68	0.1 M KOH	Ti foil	[52]
Co-Bi nanoarray	411	166	0.1 M K-Bi	Carbon cloth	[53]
Co_2_P nanoneedles	310	50	1 M KOH	Glassy Carbon	[54]
NiCo LDH	367	40	1 M KOH	Carbon paper	[55]
CoP	400	57	1 M KOH	Glassy Carbon	[56]
Pb-TCPP	470	106	1 M KOH	Glassy Carbon	[35]
Fe/Ni-BTC@NF	270	47	0.1 M KOH	Nickel foam	[36]
Co-MOF	360	89	1 M KOH	Glassy Carbon	Present work

**Table 4:** Summary of fitted EIS data for **1** and Co_3_O_4_.

Material	R_s_ (Ω)	R_ct_ (Ω)	C_dl_ (mF)
**1**	13.4	247.0	118
Co_3_O_4_	14.1	408.6	0.56
